# Silicon 3D Microdetectors for Microdosimetry in Hadron Therapy

**DOI:** 10.3390/mi11121053

**Published:** 2020-11-28

**Authors:** Consuelo Guardiola, Celeste Fleta, David Quirion, Giulio Pellegrini, Faustino Gómez

**Affiliations:** 1 CNRS/IN2P3, IJCLab, Université Paris-Saclay, 91405 Orsay, France; 2 IJCLab, Université de Paris, 91405 Orsay, France; 3 Centro Nacional de Microelectrónica (IMB-CNM, CSIC), 08193 Bellaterra, Spain; celeste.fleta@csic.es (C.F.); david.quirion@imb-cnm.csic.es (D.Q.); giulio.pellegrini@csic.es (G.P.); 4 Department de Física de Partículas, Universidad de Santiago de Compostela, 15782 Galicia, Spain; faustino.gomez@usc.es

**Keywords:** microdosimetry, hadron therapy, linear energy transfer (LET), microdosimeters

## Abstract

The present overview describes the evolution of new microdosimeters developed in the National Microelectronics Center in Spain (IMB-CNM, CSIC), ranging from the first ultra-thin 3D diodes (U3DTHINs) to the advanced 3D-cylindrical microdetectors, which have been developed over the last 10 years. In this work, we summarize the design, main manufacture processes, and electrical characterization of these devices. These sensors were specifically customized for use in particle therapy and overcame some of the technological challenges in this domain, namely the low noise capability, well-defined sensitive volume, high spatial resolution, and pile-up robustness. Likewise, both architectures reduce the loss of charge carriers due to trapping effects, the charge collection time, and the voltage required for full depletion compared to planar silicon detectors. In particular, a 3D‒cylindrical architecture with electrodes inserted into the silicon bulk and with a very well‒delimited sensitive volume (SV) mimicked a cell array with shapes and sizes similar to those of mammalian cells for the first time. Experimental tests of the carbon beamlines at the Grand Accélérateur National d’Lourds (GANIL, France) and Centro Nazionale Adroterapia Oncologica (CNAO, Italy) showed the feasibility of the U3DTHINs in hadron therapy beams and the good performance of the 3D‒cylindrical microdetectors for assessing linear energy distributions of clinical beams, with clinical fluence rates of 5 × 10^7^ s^−1^cm^−2^ without saturation. The dose-averaged lineal energies showed a generally good agreement with Monte Carlo simulations. The results indicated that these devices can be used to characterize the microdosimetric properties in hadron therapy, even though the charge collection efficiency (CCE) and electronic noise may pose limitations on their performance, which is studied and discussed herein. In the last 3D‒cylindrical microdetector generation, we considerably improved the CCE due to the microfabrication enhancements, which have led to shallower and steeper dopant profiles. We also summarize the successive microdosimetric characterizations performed with both devices in proton and carbon beamlines.

## 1. Introduction

Exposure to radiation produces a great diversity of biochemical effects in tissues. The cellular responses depend on the amount of energy deposited by the radiation as well as the pattern of energy deposition distribution in the track structures. The related ionization processes occur at the DNA scale, and therefore the biological damage might be high or even irrevocable (i.e., cell mutation or cell death). Radiation therapy (RT) is based on this premise. While RT treats about 52% of cancers [1], it may be classified as an aggressive treatment that is limited near vital organs due to the high-risk side-effects. The radiation may be delivered by a machine outside the body (external-beam radiation therapy, mainly based on photon or electron beams) or it may come from radioactive material placed in the body near cancer cells (internal radiation therapy or brachytherapy). New techniques for external-beam RT that provide treatment noninvasively have been introduced in recent years to reduce the side-effects, such as intensity-modulated radiation therapy (IMRT) and particle therapy (PT), also known as hadron therapy. PT uses protons or charged light ions as alphas and carbon nuclei at high energies, which are directly ionizing radiations [2,3]. This means that these charged particles can penetrate human tissues with limited diffusion and maximum dose deposition close to the ends of their ranges, which is characterized by a peak shape (Bragg peak) that can be positioned into the target (tumor) to be treated. This is an important advantage compared to conventional RT, which is based on photons of short wavelengths (X-rays or gamma rays). Due to the photon absorption processes and the strong scattering suffered during the interactions with matter, photon beams spread rapidly and have an undefined range. This is characterized by the photon absorption curve, where there is an initial growth in the deposited dose followed by an exponential decrease. In contrast, in PT, the shallow initial dose keeps the irradiation low in healthy tissues in the entrance and the finite range limits the radiation field to the distal part of the tumor. Thus, the main advantage of PT is that it achieves very high dose conformity around the tumor, allowing for better protection of the organs at risk (decreasing the radiation side-effects compared with conventional RT). This is extremely relevant for radio-resistant tumors that require high-dose treatments, for those localized near at-risk organs or sensitive structures (e.g., the spinal cord), and for pediatrics cancers that require a reduction in acute and long-term morbidity.

Although proton therapy was proposed by Robert Wilson more than half a century ago [4], it has been implemented slowly because cyclotron or synchrotron facilities are required and such accelerators are more complex and costly than conventional RT accelerators [5]. In the 1950s, the first clinical proton therapy facility was installed in the Lawrence Berkeley Laboratory (University of California). Since then, more than 100,000 patients have been treated with PT worldwide (85% with protons and 15% with heavier ions, mainly carbon). Currently, there are 89 proton ion and 12 carbon ion therapy facilities in operation worldwide [6].

PT provides several advantages over standard RT: (i) The Bragg peak fits into the target position very precisely using modern imaging techniques such as computed tomography and magnetic resonance scans. Since the Bragg peak is too narrow to treat extended tumor volumes, beams of different energies are superimposed to generate a spread-out Bragg peak (SOBP) to cover uniform dose distributions; (ii) it may reduce the radiation dose to nearby healthy tissue and critical organs; (iii) there is a smaller angular scattering area and penumbra, and (iv) lastly, it may deliver a more radiobiologically effective dose [3]. This last property is due to the fact that the charged particles exhibit a high ionization pattern along their tracks, and thus the energy transferred locally into cells is higher than in conventional RT, inducing complex cellular damage. These microscopic energy deposition patterns are measured in terms of radiation quality parameters such as the lineal energy of the beam (*y*) [7]. This is related to the linear energy transfer (LET), which is the macroscopic-level equivalent parameter [8,9]. LET is an average over a large number of interactions, whereas *y* quantifies a single deposition event, and thus the random fluctuations in the energy deposition. In this context, microdosimetry is the study of the spatial and temporal distributions of the energy deposited in well-defined microscopic volumes [7].

Microdosimetry spectra, which represent the fluctuations of energy deposition and the associated stochastic quantities, are given in terms of *y* as:

(1) $$y=\frac{\varepsilon }{\bar{l}}$$

This is the quotient of the energy imparted, ε, by a single event, while $\bar{l}$ is its corresponding mean chord length ($\bar{l}$). According to Cauchy’s theorem for a convex volume (such as the 3D structures herein) under µ-randomness and uniform isotropic fields,$\;\bar{l}$ is given by:

(2) $$\bar{l}=\frac{4V}{S\times \xi }$$

where *V* and *S* are the sensitive volume (SV) and area, respectively; *ξ* is the tissue equivalent (TE) conversion factor. The lineal energy values must be corrected by two correction factors, namely (i) the charge collection efficiency (CCE) and (ii) the tissue equivalence, i.e., the silicon-to-water conversion. Once the energy spectrum is obtained, it is possible to generate the probability distribution of the lineal energy, *f(y)*. Likewise, the first moment of *y* (the frequency mean lineal energy), $\bar{y_{F}}$, can be calculated as:

(3) $$\bar{y_{F}}=\int yf\left(y\right)dy$$

Once this is known, the dose‒weighted distribution, or microdosimetric dose distribution, may be expressed as a function of the lineal energy as:

(4) $$d\left(y\right)=\frac{yf\left(y\right)}{\bar{y_{F}}}$$

The mean value of this distribution is denoted by the dose mean lineal energy, $\bar{y_{D}}$, which is calculated as:

(5) $$\bar{y_{D}}=\frac{1}{\bar{y_{F}}}\int y^{2}f\left(y\right)dy=\int yd\left(y\right)dy$$

Further details about how to obtain the microdosimetry distributions can be found elsewhere [7]. Kellerer and Rossi showed the relation between the microdosimetry and corresponding radiobiology effects through the theory of dual‒radiation action [10].

From a radiobiological perspective, the parameter used to characterize a given radiation type is its relative biological effectiveness (RBE) [11]. This is defined as the ratio between a reference radiation dose (e.g., ^60^Co, γ-rays or 250 keV X-rays) and the charged particle dose that triggers the same biological effect. The RBE depends on the dose, LET (or *y*), choice of endpoint, cell line, i.a. [11]. LET varies with depth within the irradiated tissue in PT, and so does the RBE. Larger values of LET are correlated with a higher RBE before the overkilling turning point [3]. Although protons are low-LET particles, their LET values sharply increase at the end of their range. However, most proton facilities use the RBE value of 1.1 in clinical treatments [12,13], even though some in vitro tests have shown that along the distal edge of the Bragg peak, the RBE may reach 1.7 in proton therapy [13]. Proton therapy without proper RBE optimization can reduce the quality of the treatments. Including RBE models in treatments could enhance the normal tissue complication probability and decrease the tumor control probability [14,15,16,17]. In response to this issue, radiobiological optimization of proton therapy is being considered by optimizing the LET or RBE distributions [18,19]. This requires RBE model implementation based on the parameter *y* [20].

Tissue‒equivalent proportional counters (TEPCs) have traditionally been used to perform microdosimetric measurements [21,22]. However, they have some shortcomings [22]: (i) they suffer wall effects from the scattering and secondaries, (ii) need bulky readout‒electronics, (iii) require gas and a high-power supply (until 1000 V), (iv) are point-like, i.e., highly limited in spatial resolution, and (v) the associated sensor setup is large, which increases the pile‒up effects. Consequently, TEPCs are not practical for daily microdosimetry, even if the performance of new mini‒TEPCs has improved recently [23,24]. In contrast, silicon‒based radiation microdetectors can tailor the micrometer sites, they do not require gas supply, can work at a few volts, and comprise portable systems with fast response times [25,26,27,28,29,30,31]. When designing a silicon‒based microdosimeter, the sensor must have a well-defined radiation sensitive micro‒volume [7]. For this purpose, we designed and fabricated novel radiation detectors with both 3D and 3D‒cylindrical architectures, which were etched inside the silicon bulk in the National Center of Microelectronics (IMB‒CNM, CSIC, Spain). These 3D microstructures were specifically customized for microdosimetry in PT and they overcame some of the technological challenges in this domain, namely the low noise capability, well‒defined sensitive volume, high spatial resolution, and pile‒up robustness [32,33,34,35,36,37,38,39,40,41]. Both architectures reduce the loss of charge carriers due to trapping effects, the charge collection time, and the voltage required for full depletion compared to planar silicon detectors. Particularly, in the 3D‒cylindrical architecture, electrodes are placed in the silicon bulk with a very well‒delimited SV, which mimics a cell array with shapes and sizes similar to those of mammalian cells, whose diameters range from 10 to 100 µm. Other alternatives as microdosimeters are based on diamond, since it is tissue equivalent and radiation hardness [42,43]. Recent new diamond microdosimeters [44,45] have begun to explore their microdosimetric performance.

Lineal energy values in proton beams starts from 1‒2 keV/µm. Considering the ideal SV thicknesses (≤20 µm), the signal‒to‒noise ratio is a challenge. Due to this, there are few studies in the literature regarding the use of silicon‒based detectors under clinical conditions. The scarcity of publications is due to the fact that the emerging energy threshold during clinical measurements can be considerably higher than the perceptible *y* values delivered in clinical beams. For example, Rosenfeld’s group has developed silicon‒based microdosimeters over the last two decades based on planar PN junctions with implants on the front face, whose silicon boundaries are etched to avoid charge collection sharing [31]. Likewise, Agosteo et al. created a ΔE_E silicon telescope that is useful for beam characterization [30]. On the other hand, the reliable measurement of lineal energy distributions above 1 keV/µm sets a lower limit on the mean chord length of the site used for silicon devices without an intrinsic gain of around 5 µm [40]. As a consequence, solid‒state devices with the necessary low measurement threshold cannot be produced at the sub‒micrometric scale.

The present review reports on the microdosimetric characterization of both proton and carbon beams by using two novel silicon‒based 3D micodetectors created in the IMB‒CNM. These sensors allow for further RBE calculations in hadron therapy beams under clinical conditions.

## 2. Silicon‒Based 3D Microdosimeters

**Figure 1:**
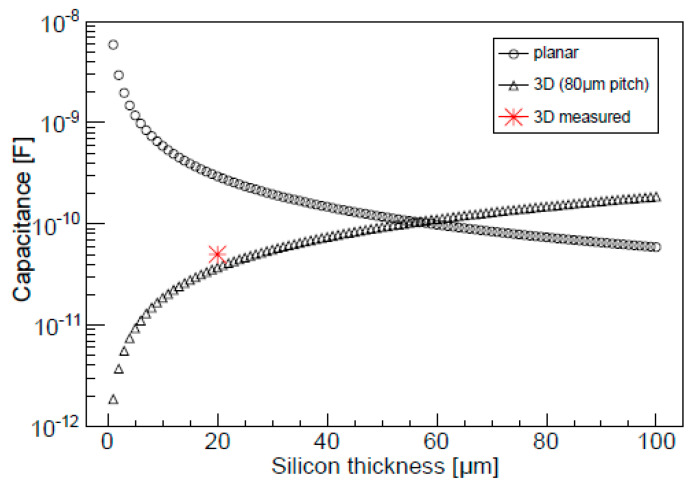
Capacitance of parallel‒plate and 3D silicon detectors versus the silicon bulk thickness for similar sensor areas. The 3D structure shows lower capacitance values for thicknesses lower than 50 µm. For a silicon thickness of 10 µm and with an 80 µm pitch (value used in the detectors for the ATLAS semiconductor tracker (SCT) at CERN), the U3DTHIN capacitance is two orders of magnitude smaller than for a planar silicon detector with the same thickness and surface area. Image taken from [36].

Standard radiation detectors have traditionally used planar technology, where electrodes are implemented on the semiconductor’s surface. In 1997, Parker, Kenny, and Segal [46] proposed an innovative design, namely *a 3D architecture for solid-state radiation detectors*, by creating columnar electrodes that penetrate into the semiconductor substrate. Based on this concept, the IMB‒CNM developed the Parker’s 3D diode over recent years for high‒energy physics experiments and medical physics applications [47,48,49,50,51,52,53]. In the 3D detectors, the depletion voltage does not depend on the silicon bulk thickness, but on electrode spacing. The electric field and the charge drift are generated perpendicular to the particle track. Therefore, both the collection distances and times can be reduced with this design and are two orders of magnitude lower than those obtained with planar technology [46]. Due to the confined electric field, there is less carrier diffusion outwards and therefore the charge sharing between adjoining electrodes is negligible [54]. Following the approximation of a coaxial cable capacitor, the associated capacitance is given as:

(6) $$C=\frac{2\pi \varepsilon L}{ln\left(\frac{r_{d}}{r_{c}}\right)}$$

where *L* is the electrode length, *r_d_* is the radius of the depleted cylindrical volume, and *r_c_* is the radius of the electrode. Figure 1 shows the capacitance of a parallel‒plate silicon detector and a 3D detector versus the silicon thickness.

In the planar geometry, the thinner the sensor, the higher the electrode‒to‒backplane capacitance, since it is inversely proportional to the thickness of the sensor, and thus the signal‒to‒noise ratio decreases. However, with the 3D geometry, the capacitance is two orders of magnitude lower than that of a planar sensor of the same thickness. Nevertheless, as the thickness increases, the 3D capacitance increases linearly with the thickness to match the planar case. This means that the 3D configuration is advantageous for thicknesses lower than 50 µm. An extensive simulation study of the electric behaviour of these detectors can be found in [55].

Hence, 3D detectors allow for lower electronic noise with thicknesses in the range of a few micrometers. This feature is particularly useful for microdosimetry in proton therapy, where the *y* values delivered are low (i.e., 1–2 keV/µm) and therefore low energy thresholds are required.

Another useful feature for 3D detectors is related to their radiation hardness, since they have been proven to work well for a fluence of 10^17^ 1 MeV neutron-equivalent particles∙cm^−2^ [56].

The Radiation Detector Group at IMB‒CNM has developed various 3D technologies for high-energy experiments over the years. One of the research lines derived from these 3D developments has been focused on creating new microdosimeters. In particular, two different types of 3D silicon microdosimeters were manufactured at IMB‒CNM. The first one belongs to the U3DTHIN architecture [35,37,50,57,58,59]. On the basis of the preliminary results with U3DTHIN detectors, a novel architecture based on 3D‒cylindrical microstructures was proposed and specifically developed for microdosimetry in hadron therapy [32,33,34,36,38,39,40,41].

### 2.1. Ultra-Thin 3D Silicon Detectors

#### 2.1.1. Microfabrication Processes

**Figure 2:**
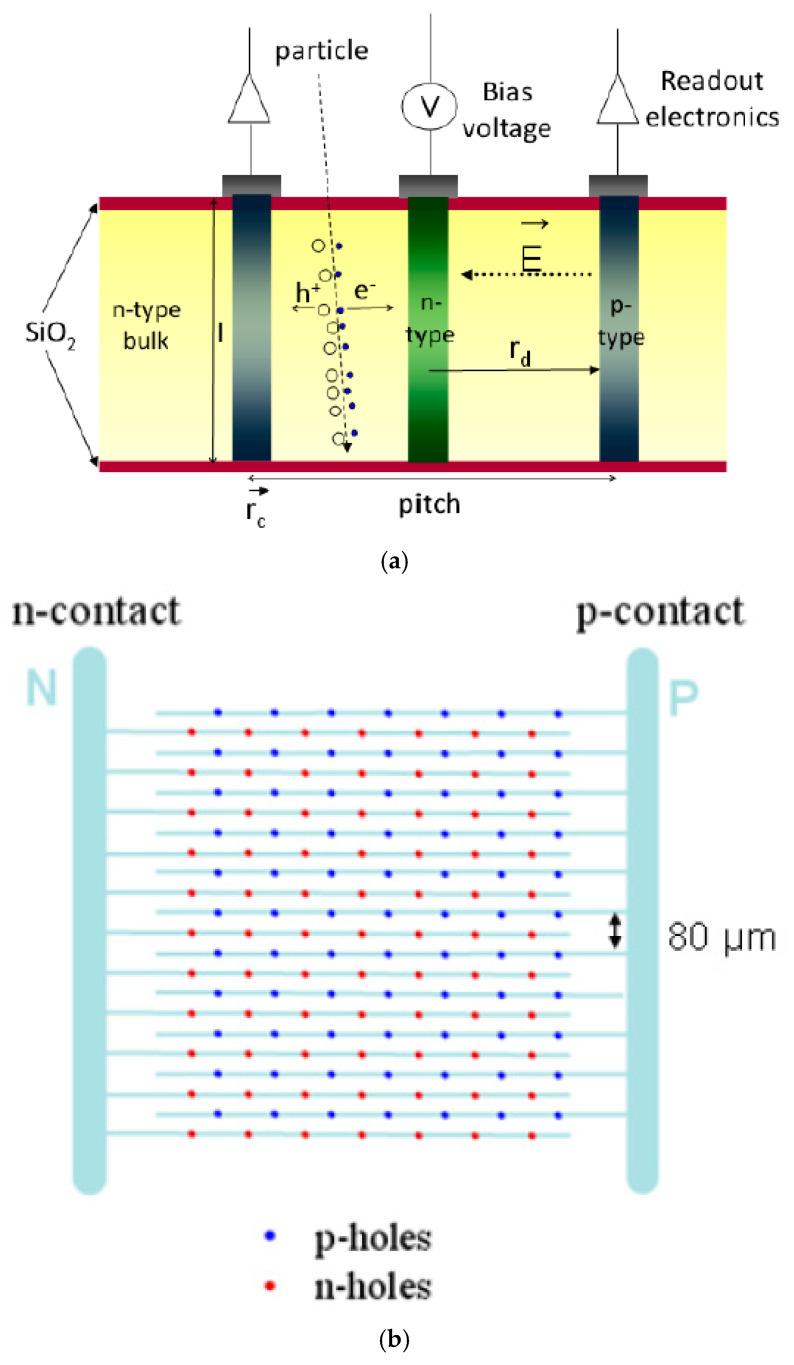
Sketch of the cross-section (**a**) and frontside (**b**) of an ultra-thin 3D diode (U3DTHIN). The layouts show the electrodes and strips that connect the p-holes and n-holes with the p- and n-contacts, respectively (this sketch is for a pad configuration, i.e., the strips are shorted to one electrode). Images taken from [50].

The first ultra-thin 3D diodes (U3DTHINs) were developed at IMB‒CNM from 2008 to 2012. They consisted of 3D columnar structures with P–N junctions fabricated on silicon-on-insulator (SOI) wafers (Icemos Technology Ltd. (Belfast, Northern Ireland); n-type wafers with 10 and 20 µm thick Si layers, 1 µm thick buried silicon oxide layer, and 300 µm thick silicon handle wafer). The handle wafer can be etched from the backside, leading to novel 3D detectors with thin membranes. Initially, these detectors were fabricated for plasma diagnostics [48,57] and neutron detection [50,58,59] in order to achieve high gamma ray rejection. This was possible thanks to their thinness and ability to discriminate the signals coming from the neutrons in mixed neutron–gamma fields in radiotherapy [58]. Figure 2 shows a sketch of the U3DTHIN.

The fabrication process starts with field oxidation, then p+ and n+ electrodes are successively etched (DRIE), filled with polysilicon, and doped with boron and phosphorous, respectively. The electrodes are inactive, and therefore are manufactured as narrowly as possible (i.e., holes of 5 µm in diameter). Then, aluminium lines are defined for interconnection and a silicon nitride–silicon oxide passivation layer is deposited. Finally, for microdosimetric applications, the handle wafer can be etched from the backside to form membranes, whose thickness is determined by the top active silicon layer.

**Figure 3:**
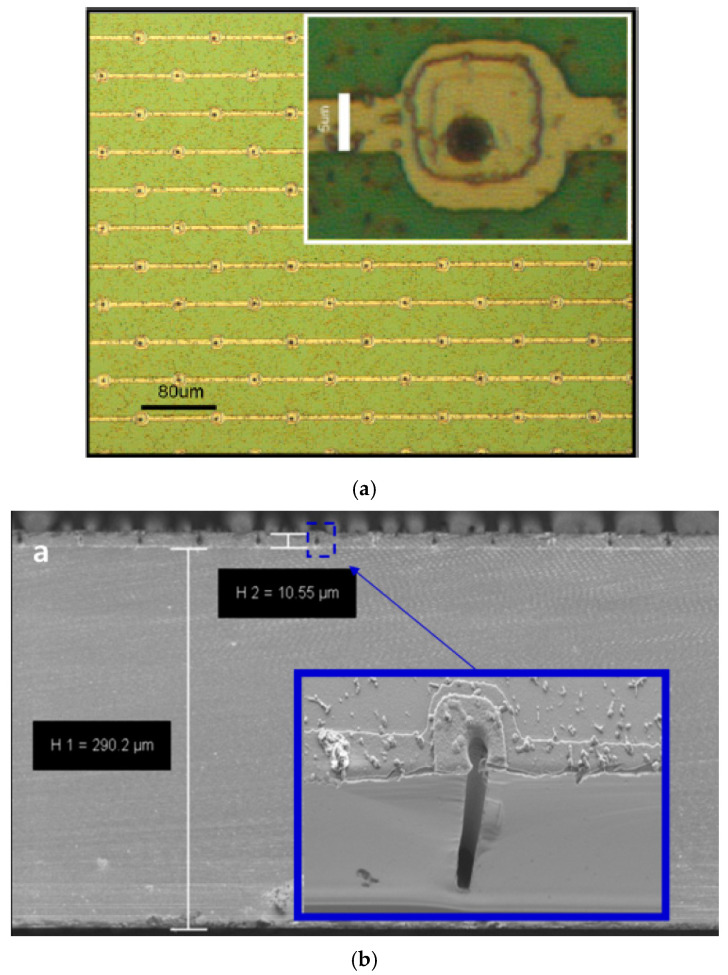
(**a**) Optical microscope image of the top of one part of a representative U3DTHIN, where the connected electrodes are shown, along with a magnified view of one of them. (**b**) Scanning electron microscopy image of the cross‒section of one U3DTHIN detector, where the columnar electrodes distributed along the surface are shown. Images taken from [50].

The columnar electrodes were distributed in a square array with an 80 μm pitch between columns of the same doping type. The whole radiation-sensitive area was 0.57 cm^2^. The full U3DTHIN fabrication processes, as well as the layouts, are described in detail in [36,50]. Figure 3 shows some representative pictures of the manufactured U3DTHINs.

Taking advantage of their thin sizes (i.e., 10 and 20 µm thicknesses), U3DTHINs were also tested to characterize their potential use in microdosimetry [35,37].

#### 2.1.2. Electrical Characterization

**Figure 4:**
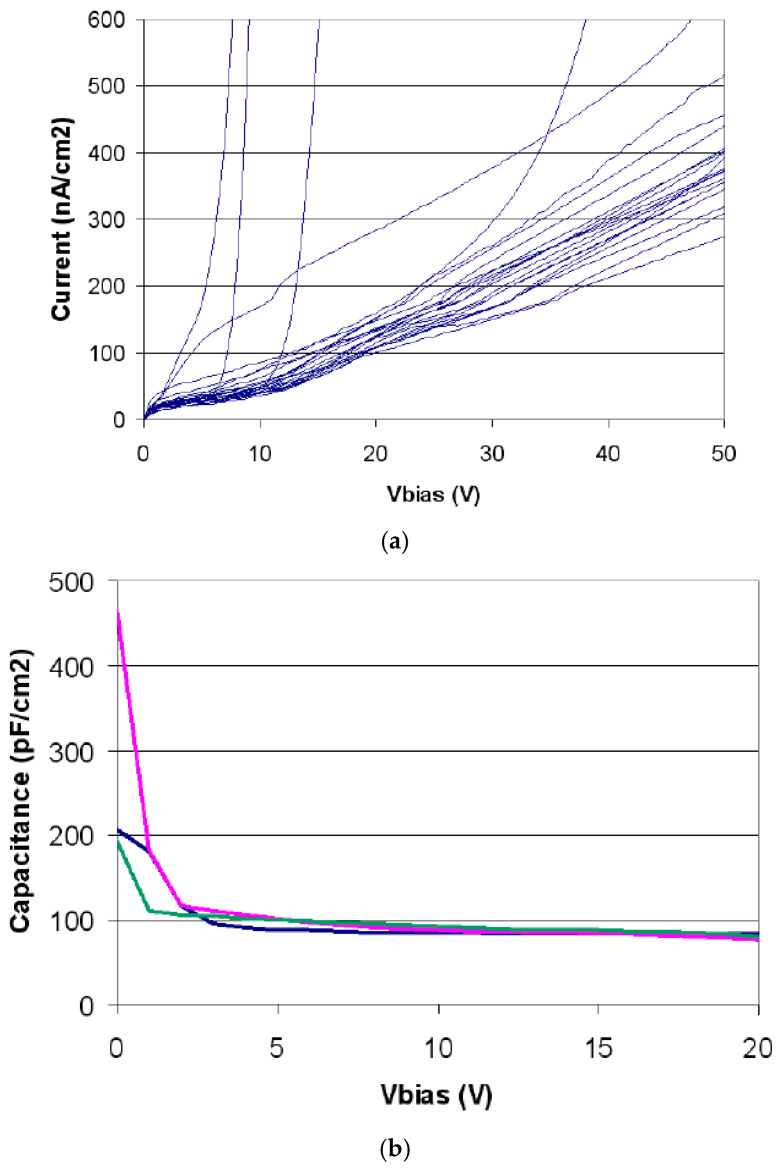
I‒V and C‒V curves of representative U3DTHINs. Images taken from [50].

Figure 4 shows two representative electrical characterization current–voltage (I‒V) and capacitance–voltage (C‒V) curves: the leakage currents were in the range of 70 ± 10 nA/cm^2^ and the capacitances were in the range of 70 ± 10 pF/cm^2^ at 10 V (lateral depletion voltage at 5 V).

#### 2.1.3. Readout Electronics

**Figure 5:**
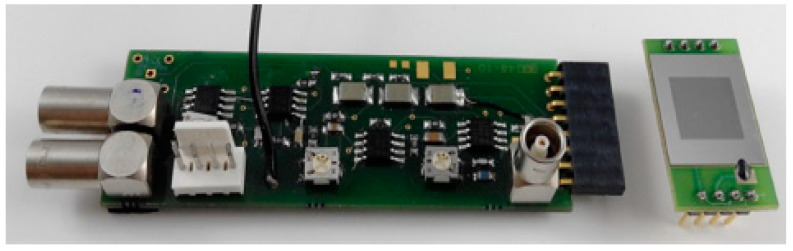
Photograph of the readout electronics: the portable electronics (left), measuring 10 cm in length, and a U3DTHIN (right) attached to an independent board, which can be connected to it. These separated boards allow the user to test different U3DTHINs with the same readout electronics. Image taken from [36].

The e^‒^‒h+ pairs created by the charged particles over the sensor have to be amplified correctly once they are collected in the electrodes. This was done using a combination of a preamplifier, shaper, and amplifier electronics [60]. The preamplifier was configured as a current integrator to convert the current pulse at a voltage large enough to be treated and adapted with the minimum noise level possible. The subsequent CR high‒pass filter introduced the desired decay time and the RC low‒pass filter limited the bandwidth and set the rise time. These two filters attenuated the signal at high and low frequencies, where there was no useful information, improving the signal‒to‒noise ratio. ADA4817 (Analog Devices, Norwood, MA, USA) ultra-high speed voltage feedback amplifiers with FET inputs were used. Figure 5 shows the portable readout electronics powered at ± 5 V. This system was combined with a multichannel pulse height analyzer MCA8000A (Amptek, Bedford, MA, USA), connected from the experimental room to the control room to a PC via Ethernet with an ADMCA display and acquisition software. An energy calibration process was performed with either an injection of electronic pulse, which simulated the sensor output signal, or with alpha sources, e.g., ^241^Am and ^238^Pu. A response of 5 V/MeV in silicon was found using a pulse‒shaping stage with a time constant of 2.5 µs.

Nevertheless, the U3DTHIN detectors had two main disadvantages: (i) the SV was not completely defined due to the open-ended pillar configuration (see Figure 2), and therefore the mean chord length might vary regarding the total sensor surface (7 mm × 7 mm), which may affect the microdosimetry spectrum; (ii) the electrode columns are an inactive volume inside the detector itself, and thus they should be fabricated to be as narrow as possible. Both restrictions are related to each other, since the aspect ratio between the diameter and the depth of the holes in the etching process is limited to around 1:30.

### 2.2. 3D‒Cylindrical Microdetectors

#### 2.2.1. Microfabrication Processes

**Figure 6:**
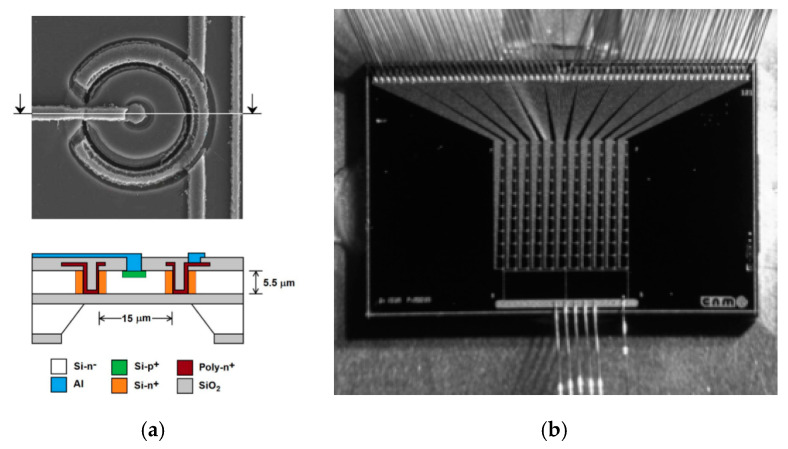
(**a**) Front face of a 3D‒cylindrical unit cell in the microdosimeter. (**b**) Photograph of a manufactured 11 × 11 microdetector array. On the top the upper fan-out connections directed towards the readout electronics are shown. On the bottom the lower part connects all of the rings surrounding the active areas to a common ground. Images taken from [38].

On the basis of the U3DTHINs’ performance, an advanced microdosimeter was designed with a novel 3D‒cylindrical architecture [32,33]. During 2012–2015, we manufactured this design, which consists of unit cells of 9–25 μm diameter with quasi‒toroid electrodes and depths of 5, 10, and 20 μm, with a well-defined micrometric cylindrical shape etched into the silicon bulk as a cell-like silicon SV. The unit cell layout was distributed as an array of independent 3D‒cylindrical microdetectors with separations between p‒electrodes (i.e., pitches) ranging from 25 to 200 µm. Figure 6 shows two representative sketches of a unit cell and a matrix of unit cells. Each unit cell works as an individual solid‒state microdosimeter.

**Figure 7:**
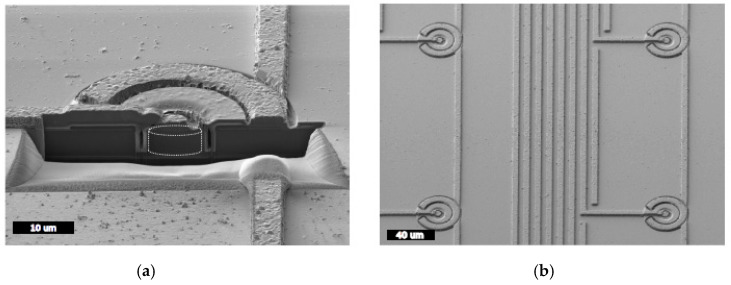
(**a**) SEM image of the cross-section of one-unit cell, which was cut using a focused ion beam machine. Note that the radiation-sensitive volume is highlighted with a white outline in a cylindrical shape. (**b**) SEM image of an array of 3D microdetectors (15 μm diameter, 5.4 μm thickness), showing the metal strips ready to be connected to an appropriate readout electronics system. Images taken from [36].

The three detector types (i.e., pads, strips, and pixel detectors) were fabricated. In all the configurations, n+ electrodes are connected together with metal lines to a n+ contact on one side of the sensor and arranged in a square matrix (e.g., 3 × 3 and 11 × 11 unit cells). Sensors were manufactured over SOI wafers measuring 6, 10, and 20 µm in thickness. The device silicon was <100>, n-type silicon doped with phosphorus and with a nominal resistivity >3 kΩ∙cm. The buried oxide and the support silicon thicknesses were 1 and 300 µm, respectively, for all wafers. The fabrication process was more complex than for U3DTHIN detectors, but followed the same strategy. Details of the fabrication process, electrical simulation, and charge collection study for the two consecutive generations of these sensors are described elsewhere [34,36,38,39,40,41]. Figure 7 shows scanning electron microscope (SEM) images of an array of these 3D microdetectors once manufactured.

The microsize of the unit cell not only increases the spatial resolution compared to TEPCs, but also may decrease the pile‒up in high‒fluence rate fields, such as those in hadron therapy (≥10^7^ particles∙cm^−2^∙s^−1^). As with the U3DTHINs, the SOI wafer support may be selectively etched to avoid backscattering contributions in particle beams.

This design overcomes some major issues, as follows: (i) the lowest energy level of detection is reduced by minimizing electronic noise; (ii) the well-delimited cylindrical configuration avoids charge sharing between neighboring unit cells and avoids diffusion; (iii) the field funneling effect is avoided using SOI wafers.

It is worth noting that the final active sensitive volume is reduced due to the internal diameter of the annulus trench being limited by the depth of the n+ diffusion. Such doping diffusion generates a highly doped region with low collection efficiency. Therefore, a charge collection efficiency (CCE) study is mandatory. This was evaluated using an ion-beam-induced charge (IBIC) map technique [34,41]. The main results are discussed in Section 2.2.3.

#### 2.2.2. Electrical Characterization

**Figure 8:**
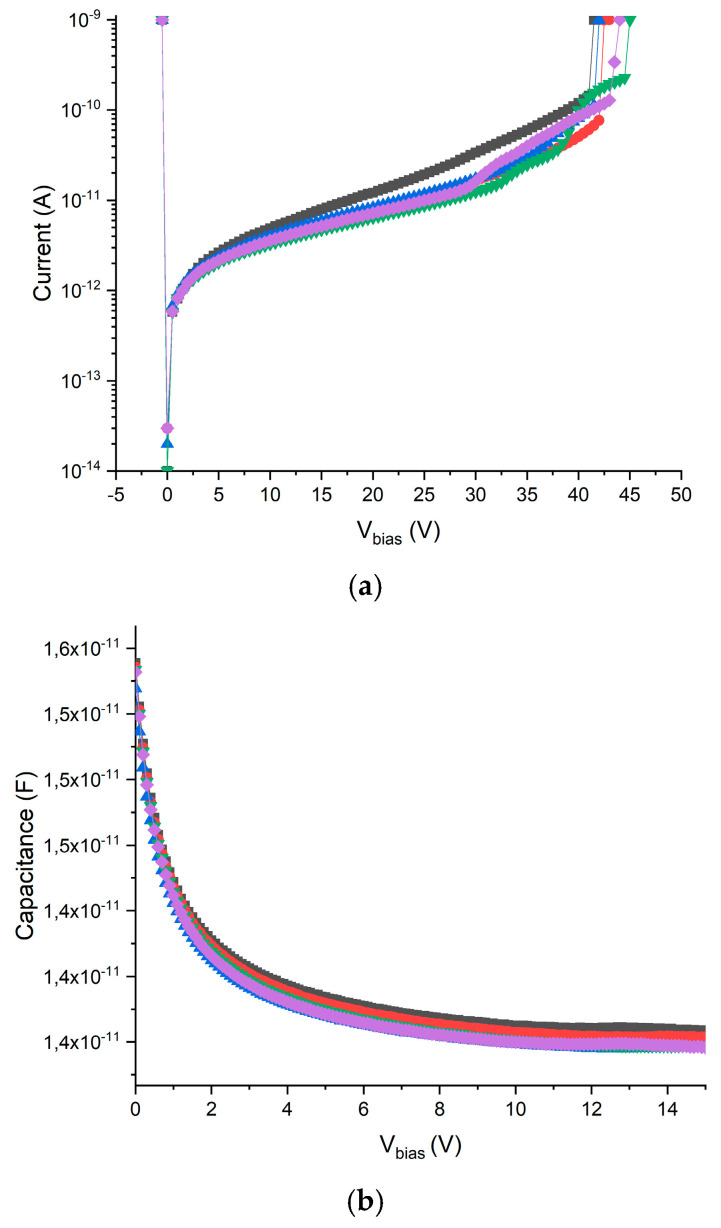
The I‒V (**a**) and C‒V (**b**) curves of 10 × 10 arrays of unit cells of 25 µm diameter and 20 µm thickness.

The 3D‒cylindrical microdetectors were tested on a wafer at a controlled room temperature of 20 °C and with an N_2_ flow to reduce humidity. As the unit cells are very small, the characterization was done in arrays of 10 × 10 cells connected to a single readout channel to increase the precision of the measurement. Figure 8 shows the current–voltage (I‒V) and capacitance–voltage (C‒V) characteristics obtained with several of these devices with cells of 25 μm diameter and 20 μm thickness. The arrays show good diode characteristics, with breakdown voltages higher than 40 V and reverse currents of 40 fA/cell at 10 V. The depletion capacitance measured at 10 kHz was 14 fC/cell. Regarding the cell matrix, the total capacitance was one order of magnitude lower than for planar sensors of equivalent thickness (see Figure 1).

#### 2.2.3. CCE Characterization

The charge collection efficiency (CCE) values for both 3D-cylindrical microdosimeter generations were studied in the National Accelerator Center (CNA, Seville). The ion‒beam‒induced charge (IBIC) technique was used in a microprobe beamline [61]. IBIC is a scanning microscopy technique in which ion beams of several MeV are launched to assess the charge collection ability of the sensors over which those beams impinge. Sensors were placed inside a vacuum chamber during the irradiation process.

The IBIC characterization of the first‒generation 3D-cylindrical microdosimeter was performed with 1 MeV protons and 2 and 5 MeV He^2+^ ions. The lower level discrimination of the multichannel analyzer was as high as 400 keV because of the experimental noise, which was significantly reduced in the second improved generation. For the second‒generation microdosimeter, IBIC tests were performed with 3.5 and 5 MeV He^2+^ ions. The studied IBIC showed an intrinsic efficiency of 100% for radial distances of up to r_eff_ = (12.26 ± 0.16) µm, with unit cells measuring 20 µm in thickness, corresponding to relative active volumes of 96.2 ± 0.6% with respect to the nominal design [41]. This result shows an important improvement with respect to the first generation, for which it had been estimated that the effective radius was reduced by 2.5 µm and the corresponding active volume was only 56% [34]. Several improvements were made to this second‒generation microdosimeter, in particular the reduction of the overall thermal budget, especially of the ohmic N^+^ contact doping, which allowed for shallower and steeper dopant profiles to be obtained. This had a significant impact in terms of the CCE improvement. The particles arriving in this low-CCE region of the detector will give rise to events in the low‒energy region of the measured spectrum. This IBIC characterization allowed us to add the corresponding CCE correction factor in the later experimental spectra. The effects of CCE could be seen alternatively as a modification of the effective chord length distribution that can be obtained from the nominal geometry of the sensor.

#### 2.2.4. Readout Electronics

**Figure 9:**
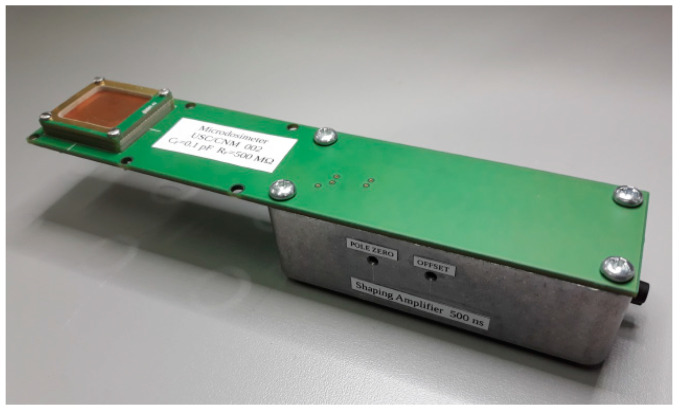
Photograph of a 3D-cylindrical microdetector setup. The dimensions of the setup (170 × 35 mm) make it a portable system. Image taken from [39].

Two readout electronics instruments were developed for the 3D-cylindrical microdetectors. In the first tests, they were connected to a Costruzioni Apparecchiature Elettroniche Nucleari S.p.A. (CAEN, Viareggio, Italy) A1422H Hybrid charge-sensitive preamplifier with a CAEN N968 spectroscopy shaping amplifier. Similarly to the readout electronics used with U3DTHINs above, we used an Amptek MCA8000D multichannel analyzer to digitize the pulse height. In the second tests, a new electronics method was performed over two different boards: one housed the detector and charge preamplifier far away (10 cm) from the second board, which housed the shaping and amplification stages. The preamplifier was an OPA657 (Texas Instruments, Dallas, USA), which is suitable for the very-low level signals. The amplifier was a fixed-gain inverter (HFA1112) combined with a CREMAT CR-200 Gaussian-shaping amplifier and a CREMAT CR-210 baseline restorer (Cremat Inc, West Newton, MA, USA), followed by an HA-5002 current buffer amplifier (Intersil, Milpitas, CA, USA) to drive the output signal. Figure 9 shows a picture of the last 3D-cylindrical microdetector setup, including the readout electronics system, which was used for assessment of a single unit cell.

## 3. Microdosimetry Results

Tests were performed with two of the significant PT particles, i.e., proton and carbon beams, the results of which are shown below.

### 3.1. U3DTHINs

**Figure 10:**
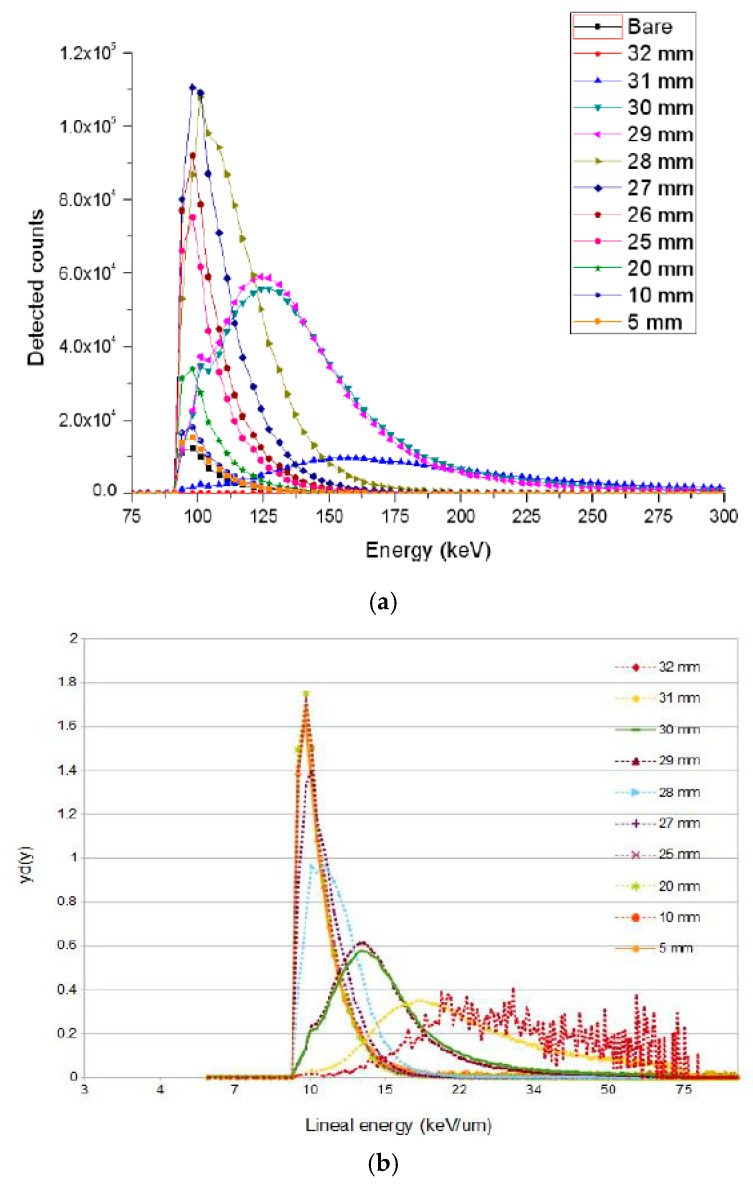
(**a**) Pulse height spectra versus the water-equivalent (P2251) thicknesses. Distributions were normalized to the fluence rate measured by a monitor chamber (10^4^ p∙cm^−2^s^−1^). (**b**) Corresponding silicon microdosimetry spectra. Images taken from [35].

U3DTHINs were connected to a readout electronics system, as explained in Section 2.1.3. First, the second improved U3DTHIN generation was tested in the CYCLONE-110 cyclotron at the Center de Recherches du Cyclotron (CRC) in Louvain-la-Neuve, Belgium. Proton beams of 62 MeV were used at the cyclotron exit. The sensors were positioned perpendicular to the particle beams. P2251 virtual water layers were used (with thicknesses of 1 to 10 mm) to obtain several depths along the corresponding Bragg curve. The readout electronics system was placed in a Faraday cage to reduce noise contributions. Figure 10a shows the pulse height spectra measured versus the P2251 thickness along the Bragg curve. The low-level discrimination threshold (LLD) was fixed at 75 keV in silicon, the details of which can be found elsewhere [35]. Figure 10b shows the microdosimetry measurements derived from the energy spectra at various depths along the Bragg curve.

Secondly, the same U3DTHINs and readout electronics were used to perform a test in a 94.98 AMeV ^12^C ion beam at the GANIL cyclotron facility (Caen, France). The average fluence rate was 2.4 × 10^4^ s^−1^cm^−2^ and the beam profile had a FWHM value of 7 mm at the beam exit, which provided uniform irradiation on the detector. The range of the 94.98 AMeV ^12^C beam was 20.5 mm in lucite. The LLD was set to 200 keV in silicon. In this case, a customized phantom system was manufactured and used for precise positioning. This consisted of a motorized remote-controlled lucite (1.186 g∙cm^−3^) wedge (10° angle) that provided continuously variable thicknesses ranging from 3 mm up to 30 mm, with uncertainty around 30 μm.

**Figure 11:**
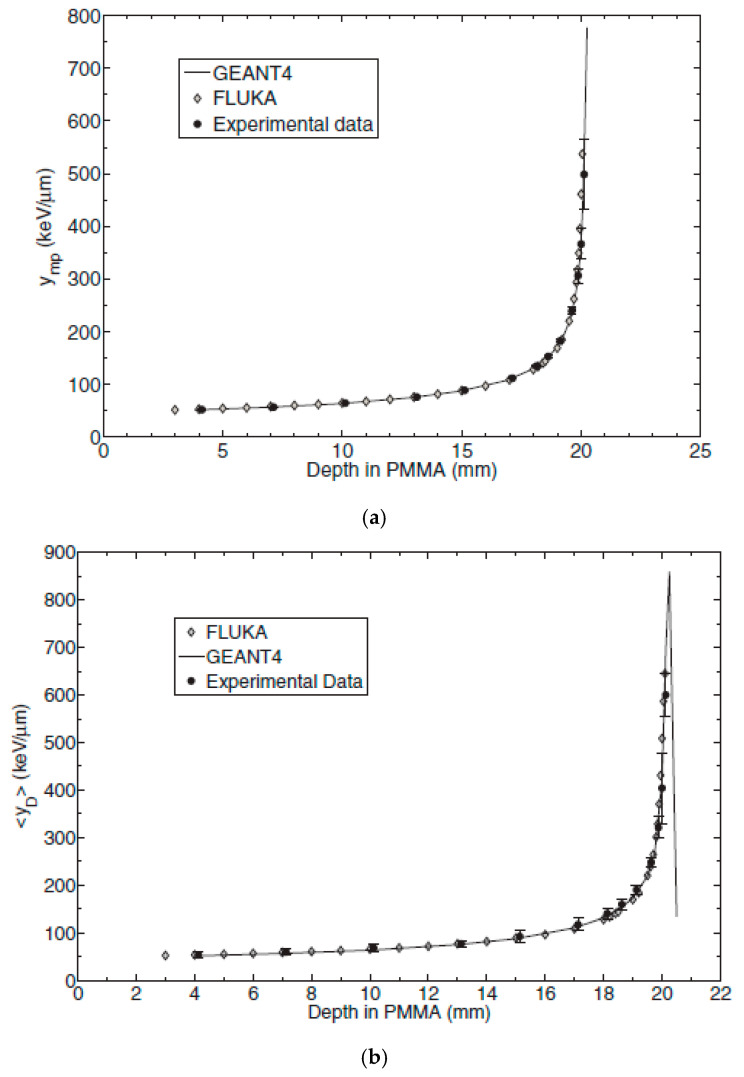
(**a**) Comparison of the most probable lineal energy values measured (circles) and those simulated with FLUKA (diamonds) and GEANT4 prediction (solid line). (**b**) Comparison of the dose-averaged lineal energy values measured (filled circles) and those simulated with FLUKA (diamonds) and GEANT4 (continuous line). Images taken from [37].

Figure 11 shows the most probable lineal energy values obtained with U3DTHINs (circles) and a comparison with two Monte Carlo codes, namely FLUKA (diamonds) and GEANT4 (solid line). The agreement between the experimental data and the simulated values was better than 4% for GEANT4 and even lower for FLUKA. Further details can be found elsewhere [37].

In light of these results, there was still room for improvement regarding the delimitation of the SV, reduction of noise, and spatial resolution. The novel design is detailed in Section 2.2 and the microdosimetry performance is show below.

### 3.2. 3D‒Cylindrical Microdetectors

The first tests were performed in the Centro Nazionale di Adronterapia Oncologica (CNAO) (Pavia, Italy) by using a 115.23 AMeV ^12^C pencil beam at a therapeutic beam fluence rate. This had a range of 28.47 mm in lucite (the water-equivalent of the material used). The beam had a diameter of 20 mm and a Gaussian profile, with FWHM values of 5.1 and 8.5 mm along the horizontal and vertical axes, respectively, at the nozzle. The average fluence rate was 5 × 10^7^ s^−1^cm^−2^, as associated with clinical beams.

The first 3D-cylindrical microdetector had a diameter of 15 μm and a thickness of 5.5 μm. A single unit cell was connected to the readout electronics shown in Section 2.2.4, which had an energy resolution of 12% at an imparted energy of 660 keV.

**Figure 12:**
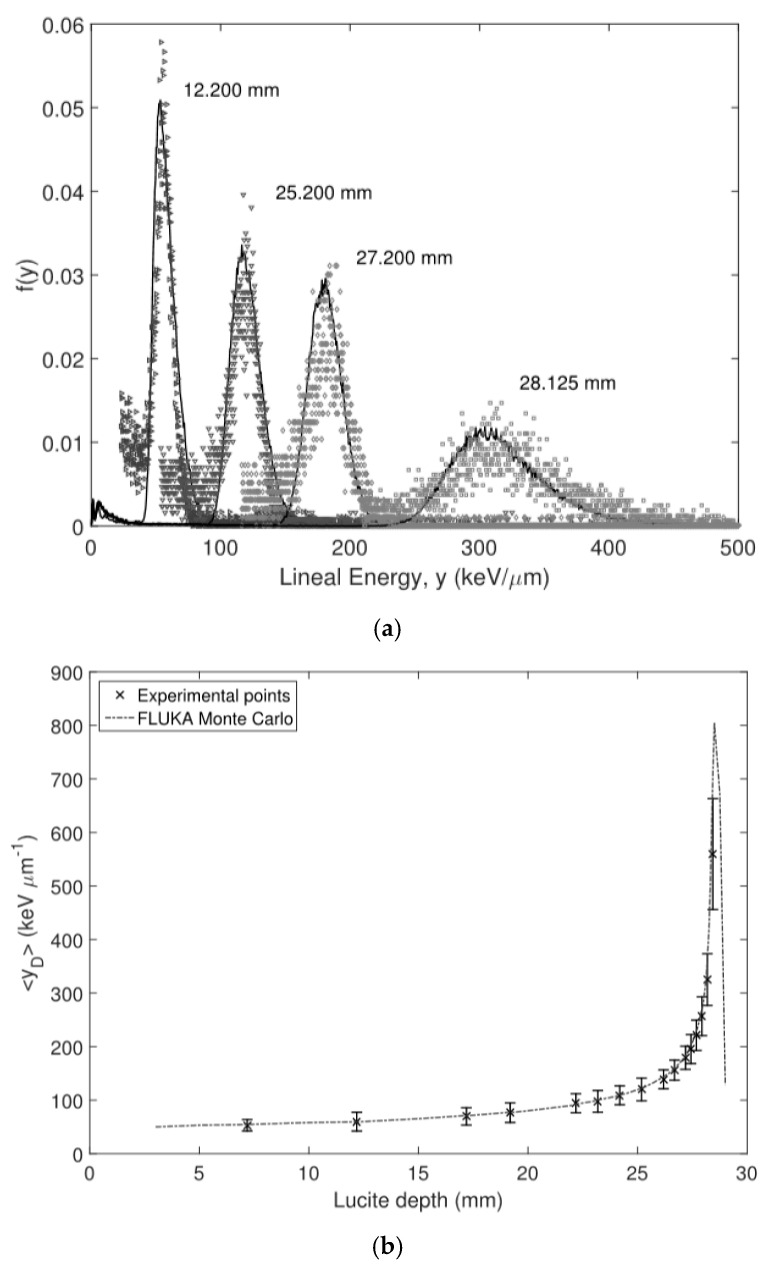
(**a**) Comparisons between the experimental and simulated lineal energy frequency distributions for different depths of PMMA across the Bragg curve. (**b**) Dose-averaged lineal energy comparison between the experimental data (crosses) and FLUKA Monte Carlo simulations (solid line). Images taken from [38].

Similarly to the case above, the Bragg curve was obtained by interposing the same lucite wedge system with submillimeter steps between the beam and the detector. Microdosimetric spectra of the lineal energy were measured at different depths up to the Bragg peak. The results were then compared with Monte Carlo simulations using the FLUKA particle transport code, showing an excellent agreement between experimental and simulated microdosimetric distributions. The agreement between experimental data and simulations was evaluated using a gamma test. The gamma index values were lower than 1 overall [38]. Figure 12 shows the dose-averaged lineal energy comparison between the experimental data and the simulations.

The microdosimetric spectra generally showed a good agreement with the Monte Carlo outcomes. These results indicate that silicon 3D-cylindrical microdetectors can be used to characterize the microdosimetric and radiobiological properties of clinical beams in hadron therapy.

## 4. Discussion

Silicon‒based radiation detectors can overcome many of the disadvantages of TEPCs, e.g., they do not require a gas supply, have fast response times, and high spatial resolution, and work at low voltages. Following appropriate tissue correction, they have contributed significantly to microdosimetry verification in recent years, mainly thanks to an Australian and two European research groups. In the first four generations of microdetectors developed by the Australian group [29,31], the silicon microdosimeters were mainly based on planar PN junctions with implantations on the front face, whose silicon boundaries were etched afterwards to avoid charge collection sharing. Their arrays were divided into segments to reduce the capacitance noise. In the last generation, a similar configuration to our proposed 3D-cylindrical microstructures was recreated in a clean-room facility in Norway [62]. In Europe, on the one hand, Pola et al. [63] recently proposed a telescope detector with a matrix of pixels (2 μm in thickness) coupled with a deeper stage (about 500 μm in thickness) based on the previous design from Agosteo et al. [30]. This design suffers from partial charge collection, which affects 10% of the total absorbed dose, however good microdosimetry performance is expected. On the other hand, to the best of our knowledge, the first 3D‒cylindrical microstructures etched into the silicon bulk were created at IMB‒CNM in 2012‒2015 [32,33,34,36] and later improved and characterized with clinical beams [38,39,40].

Regarding the U3DTHINs, the tests at GANIL (Section 3.1) showed the feasibility of the use of 3D diode silicon structures for the measurement of the microdosimetric distributions of ion beams. However, U3DTHINs had some issues considering their response dynamical range and pile‒up.

Regarding the 3D‒cylindrical microdetectors, the test at CNAO (Section 3.2) showed the good performance of the microdetectors for assessing microdosimetric distributions in hadron therapy. The device was able to analyze linear energy distributions of clinical beams, allowing the calculation of RBE values and the use of hadron therapy beams in clinical conditions, with a fluence rate of 5 × 10^7^ s^−1^ cm^−2^ without saturation. The dose-averaged lineal energy values showed a generally good agreement with Monte Carlo simulations. The RBE values were calculated using a microdosimetric kinetic model (MKM) from the transformation of imparted energy in silicon to obtain the biological dose. The results indicated that these devices can be used to characterize the microdosimetric and radiobiological properties in hadron therapy, even though the CCE and electronic noise may pose limitations on their performance. The intrinsic field gradients and charge diffusion in the SV may have an effect on the recorded spectra, which in turn may modify the microdosimetric spectra, producing an artificial enhancement of the low lineal energy region. Electrical simulations using TCAD and proton beam IBIC tests were performed to study the active volume inside the SV. An analytical model for the CCE was performed to reproduce these effects successfully. In the second 3D-cylindrical microdetector generation, the CCE was considerably improved due to the microfabrication enhancements, mainly the reduction of the thermal budget of the N^+^ Ohmic contacts, leading to shallower and steeper dopant profiles. The CCE values ranged between 100% and 90% for radial distances up to 10.75 µm from the center of the device (for a 3D‒cylindrical microdetector of 25 µm diameter).

## 5. Conclusions

In this overview, we summarize two novel solid-state microdetector designs as well as the hadron beamline characterizations performed with them. These designs were based initially on the 3D architecture proposed by Parker et al. and on a new 3D-cylindrical design with sizes similar to those of cellular nuclei.

Currently, we are working on three-axis to further preclinical tests. First, we have already designed the third 3D‒cylindrical microdetector generation with new layouts to cover an area measuring several centimeters, which we are characterizing. Second, we are customizing a portable multichannel readout system for such microdetector multiarrays. Third, we are performing an automated software for data analysis in real time to provide a clinical friendly version of the DAQ. We are also going to perform studies on sensor stability over time and radiation hardness.

In short, 3D‒cylindrical microdetectors can have a positive impact in treatments by reducing the radiobiological uncertainties in the normal tissue surrounding the target by allowing for further RBE calculations under clinical conditions. Additionally, these sensors can be employed in the use of accelerators and radiation protection for spacecraft.
